# The Improvement of Experimentally Induced Gastric Ulcers in Rats by Inhibiting Vascularization Through the Blocking of the TNF‐α Type 1 Receptor

**DOI:** 10.1002/iid3.70279

**Published:** 2025-10-15

**Authors:** Abdullah Alattar, Reem Alshaman, Fawaz E. Alanazi, Omar Bahattab, Hanan M. Hassan, Mohammed M. H. Al‐Gayyar

**Affiliations:** ^1^ Department of Pharmacology and Toxicology, Faculty of Pharmacy University of Tabuk Tabuk Saudi Arabia; ^2^ Department of Biology, Faculty of Science University of Tabuk Tabuk Saudi Arabia; ^3^ Department of Pharmacology and Biochemistry, Faculty of Pharmacy Delta University for Science and Technology Gamasa City Egypt; ^4^ Department of Biochemistry, Faculty of Pharmacy Mansoura University Mansoura Egypt; ^5^ Department of Pharmaceutical Chemistry, Faculty of Pharmacy University of Tabuk Tabuk Saudi Arabia

**Keywords:** extracellular signal‐regulated kinase (ERK), gastric ulcer (GU), intercellular adhesion molecule (ICAM)‐1, phosphatidylinositol‐3 kinase (PI3K), protein kinase B (PKB, AKT), tumor necrosis factor‐α type 1 receptor (TNFR1)

## Abstract

**Background:**

About 5%–10% of the world's population is affected by gastric ulcers, which can result in gastrointestinal perforation and bleeding. Consequently, we aimed to investigate whether blocking TNF‐α type 1 receptor (TNFR1) with CAY10500 could diminish experimentally induced gastric ulcer (GU) in rats by modulating vascularization.

**Methods:**

Rats were administered with a single oral dose of 80 mg/kg of indomethacin to produce gastric ulcers. Subsequently, some rats were given 1 mg/kg of CAY10500 orally. Gastric samples were used to assess the genetic expression and protein levels of TNFR1, VEGF, ERK, PI3K, AKT (also known as PKB), and ICAM‐1. Gastric sections underwent electron microscopic examination and were subjected to hematoxylin and eosin staining and immunostaining using anti‐TNFR1, anti‐VEGF, and anti‐ICAM‐1 antibodies.

**Results:**

CAY10500 demonstrated the ability to inhibit the expression of TNFR1. Examination of micro‐images of GU using electron microscopy or H/E staining revealed extensive necrosis, resulting in the complete loss of regular ultrastructural features of epithelial nuclei and cytoplasmic organelles, as well as the loss of tight junctions and disruption of cell membranes. Significantly, the administration of CAY10500 mitigated these effects. Furthermore, CAY10500 significantly elevated the expressions of VEGF, ERK, PI3K, and AKT, which was associated with a significant reduction in the expression of ICAM‐1.

**Conclusion:**

CAY10500 effectively improved experimentally induced GU in rats. It works by inhibiting TNFR1 and activating angiogenesis and cell proliferation pathways, leading to gastric tissue healing. CAY10500 significantly reduced the adhesion molecule pathways.

## Graphical abstract

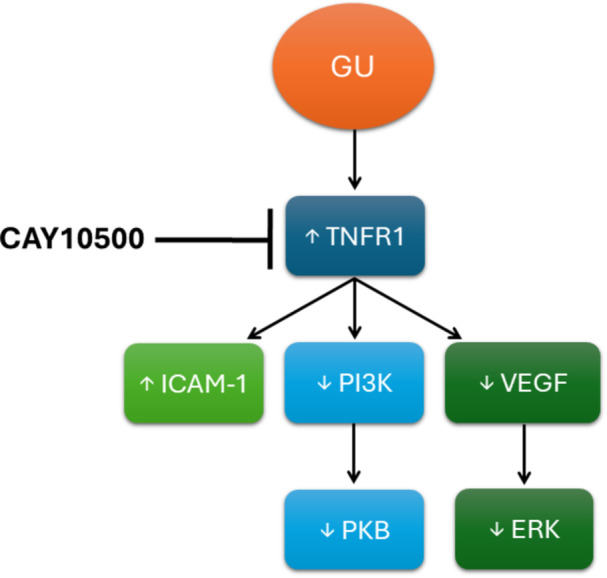

## 1 Introduction

Gastric ulcer (GU) is a condition that causes inflammation in the stomach lining. Immune‐mediated factors or infectious agents can cause it. Common symptoms include discomfort in the upper abdomen and feeling nauseous. Usually, these symptoms are concentrated along the lesser curvature and extend more profoundly than the mucosa [1]. An intricate healing process occurs in the body, including cell migration, proliferation, neovascularization, and extracellular matrix deposition. These processes work together to promote tissue regeneration around the ulcer scar. Various factors, such as cytokines, growth factors, and hormones, regulate the healing process [2]. GU affects four million people worldwide annually and has an estimated lifetime prevalence of 5%−10% in the general population [3]. The main factors responsible for gastric ulcers are infection with *Helicobacter pylori*, increased gastric acid production, and long‐term use of nonsteroidal anti‐inflammatory drugs (NSAIDs). The typical medical approach for treating peptic ulcers, including gastric ulcers, involves a therapy consisting of a proton pump inhibitor and bismuth along with two antibiotics (clarithromycin and amoxicillin) [4]. A thorough comprehension of the stomach lining's defensive mechanisms could expedite the development of innovative antiulcer therapies and contribute to the effective management of GU disease progression.

The widely recognized pro‐inflammatory cytokine tumor necrosis factor‐α (TNF‐α) has been found to play a crucial part in several mechanisms linked to the formation of stomach ulcers. Studies suggest that TNF‐α is essential in developing indomethacin‐induced GU by triggering immediate inflammatory reactions and promoting the migration of neutrophils [5]. Furthermore, TNF‐α has been linked to NSAID‐induced gastropathy, which can trigger proapoptotic caspases, leading to the apoptosis of gastric epithelial cells in animal models treated with NSAIDs [6]. Moreover, TNF‐α is known for its ability to hinder the natural healing process of ulcers by exerting inhibitory effects on gastric microcirculation, cell proliferation, and angiogenesis at the periphery of the ulcer. Additionally, the phosphatidylinositol 3‐kinase – protein kinase B (PI3K‐PKB) signaling pathway and the TNF‐α pathway can activate the apoptosis signaling pathway downstream [7]. Research has shown that increased TNF‐α levels result in the overproduction of PKB (AKT), vascular endothelial growth factor (VEGF), and intercellular adhesion molecule‐1 (ICAM‐1) [8]. The significant involvement of TNF‐α in these physiological processes highlights its importance in the formation and resolution of GU. Consequently, reducing TNF‐α levels may be a promising strategy for healing ulcers.

CAY10500 is a selective inhibitor of TNF‐α that effectively obstructs its interaction with TNF Receptor 1 (TNFR1). TNF‐α operates as a trimer, a structure that facilitates the trimerization of its receptors, subsequently activating pro‐inflammatory and apoptotic signaling cascades [9]. By specifically binding to the biologically active trimeric form of TNF‐α, CAY10500 promotes the rapid displacement of one subunit from the trimeric complex. This mechanism effectively leads to the inactivation of TNF‐α, interrupting its pro‐inflammatory signaling [10]. CAY10500 presents a targeted approach for treating inflammatory diseases. This approach can mitigate side effects compared to the general TNF‐α blockade, which may influence other critical signaling pathways. The capacity of CAY10500 to selectively inhibit TNF‐α/TNFR1 signaling has been explored in inflammatory diseases and specific cancers [11]. Our primary objective in this study was to investigate the potential impact of employing CAY10500 to inhibit TNFR1 and reduce experimentally induced GU in rats by observing its effect on mucus production and gastric tissue structure, as examined through microsections under an electron microscope and other microsections stained with hematoxylin and eosin. Next, we will examine the molecular mechanism of the protective effects of CAY10500 on GU by assessing the gene expression and gastric protein levels of VEGF, PI3K, AKT, ICAM‐1, and extracellular signal‐regulated kinase (ERK).

## 2 Materials and Methods

### 2.1 Animals and Treatment Outlines

Forty Sprague Dawley rats with a weight range of 180–200 g were used. These rats were acquired from the university's animal facility without undergoing any prior procedures. They were acclimatized to a stable environment with a 12‐h light‐dark cycle for 5 days before the commencement of the study. Before the initiation of the study, the research protocol was officially approved by the Research Ethics Committee of the Faculty of Pharmacy at Delta University for Science and Technology (FPDU‐REC) under approval number FPDU19/2022. The rats had ad libitum access to food and water throughout the experiment. They were divided into four groups, each of ten rats housed in cages designed for two rats. All treatments were administered to the rats by the same team member and were applied in the same sequence during the morning.

#### 2.1.1 Control Group

Ten rats were subjected to a 24‐h fasting period with access to water. Subsequently, they received oral administration of 0.5% carboxymethyl cellulose (CMC) via oral gavage and were not subjected to further treatment throughout the experiment.

#### 2.1.2 Control Treated With CAY10500

The rats received CMC treatment during the initial phase, followed by 1 mg/kg IV CAY10500 (Santa Cruz Biotechnology Inc., Dallas, Texas) twice weekly.

#### 2.1.3 GU Group

The experiment involved subjecting the rats to a 24‐h fasting period with access to water, followed by oral administration of 80 mg/kg of indomethacin to induce GU [12, 13].

#### 2.1.4 GU Treated With CAY10500

After the induction of GU as done in the GU group, the rats received an oral dose of 1 mg/kg of CAY10500 once daily for seven consecutive days. This treatment commenced 1 day after the administration of indomethacin to the rats [11].

### 2.2 Sample Collection

Thiopental sodium was administered at a dosage of 40 mg/kg via intraperitoneal injection to induce anesthesia in the rats. Subsequently, whole blood samples were obtained from the retro‐orbital plexus and centrifuged at 3000 rpm for 5 min to produce serum. The rats were euthanized through cervical dislocation, following which the entire stomach was removed. A section of the stomach was carefully resected, sliced, and preserved in a 10% (w/v) buffered formalin solution for subsequent morphological examination. At the same time, another section of the stomach was homogenized in a 1:10 w/v sodium‐potassium phosphate buffer solution (pH 7.4), and the resulting mixture was stored at ‐80°C until they are used for analysis.

### 2.3 Examination of Microsections by Transmission Electron Microscopy

Stomach specimens larger than 1 mm³ were carefully separated and exposed to glutaraldehyde at 4°C for 4 h to maintain the structural integrity. Following this, the specimens underwent a dehydration process using a precisely calibrated series of ethanol and propylene oxide before being carefully embedded in epoxy resin to ensure the maximum preservation of their structure. For the examination of ultrathin sections, the microsections were meticulously observed at 160 kV using a JEOL JEM‐2100 electron microscope at the Electron Microscope Unit, located at Mansoura University in Egypt.

### 2.4 Morphological Analysis and Immunohistochemistry

Gastric sections preserved in 10% formalin were processed according to standard histopathological techniques, subsequently embedded in paraffin blocks, and sectioned to a thickness of 5 µm. The sections were stained with hematoxylin and eosin. Each section was assigned an anonymous code and evaluated blindly using a digital camera‐based imaging system (Nikon Corporation). For immunohistochemistry, the paraffin sections, also 5 µm thick, were subjected to treatment with monoclonal antibodies targeting TNFR1, VEGF, and ICAM‐1 (Sigma Aldrich Chemicals Co). A counterstain of hematoxylin was applied to the sections. The sections were examined using a digital camera‐assisted computer system from Nikon Digital Camera, Japan [14, 15, 16, 17].

### 2.5 Enzyme‐Linked Immunosorbent (ELISA) Assay

The investigation used ELISA kits that were commercially available from MyBioSource, Inc. (San Diego, California, United States). These ELISA kits were used to assess TNFR1, VEGF, ERK, PI3K, AKT, and ICAM‐1 levels. The tests were carried out in strict accordance with the manufacturer's instructions. The results were analyzed using a Spectro UV‐VIS Double Beam PC Scanning Spectrophotometer by Labomed Inc. (Los Angeles, California, United States).

### 2.6 Quantitative Real‐Time Polymerase Chain Reaction (RT‐PCR)

Using established methodologies, we assessed the gene expression levels of TNFR1, VEGF, ERK, PI3K, AKT, and ICAM‐1 mRNA in rat gastric lysate [18, 19, 20, 21, 22]. Total RNA was extracted utilizing the RNeasy Mini kit (Qiagen, USA), with the concentration assessed using Maxima SYBR Green/Fluorescein Master Mix (Fermentas, USA). Subsequently, 1 µg of RNA was reverse‐transcribed into complementary DNA (cDNA) employing the QuantiTect Reverse Transcription Kit (Qiagen, USA). The expression levels of mRNA for TNFR1, VEGF, ERK, PI3K, AKT, and ICAM‐1 in rat gastric lysates were quantified using Maxima SYBR Green/Fluorescein qPCR Master Mix in conjunction with the Rotor‐Gene Q (Qiagen, USA). The primer set used in the study was summarized in Table 1. The thermal profile of RT‐qPCR is reverse transcriptase 1 cycle at 55°C for 10 min, followed by enzyme inactivation at 59°C for 2 min, then forty cycles of amplification using 95°C for 10 s, 55°C for 10 s, and finally 72°C for 30 s. Final extension is done at 72°C for 5 min. For housekeeping and internal referencing, rat β‐actin was utilized. The outcomes of the RT‐PCR analysis were represented as Cycle threshold (Ct) values. The PCR data sheet provides the Ct values for the genes of interest relative to the reference housekeeping gene, β‐actin. A control sample was included to evaluate the gene expression of each specific gene. The Relative Quantification (RQ) for each target gene was computed and standardized to the housekeeping gene using the delta‐delta Ct (ΔΔCt) method. The RQ of each gene was determined with the formula $2^{-∆∆C_{t}}$.

**Table 1:** The primers set used for detection of gene expression in rats.

Name	Sequence		Reference sequence
*β‐actin*	Forward	5′‐CCCGCGAGTACAACCTTCTT‐3′	NM_031144.3
	Reverse	5′‐CTAGTCTCCCTCCCTCAGG‐3′	
*TNFR1A*	Forward	5′‐CCAAGTGCCACAAAGGAACC‐3′	NM_013091.2
	Reverse	5′‐GTGCCTTTATCACACACCTCG‐3′	
*VEGFA*	Forward	5′‐CCAGGCTGCACCCACGACAG‐3′	NM_001110333.2
	Reverse	5′‐CGCACACCGCCATTAGGGGCA‐3′	
*ERK*	Forward	5′‐CCCCTTCGAGCATCAAACCT‐3′	NM_017347.3
	Reverse	5′‐TTCTCATGGCGGAATCCGAG‐3′	
*PI3K*	Forward	5′‐TTACGGCGGCATGGGAATCT‐3′	XM_017595947.2
	Reverse	5′‐CCAGCTTTCCCTGAGTGCCT‐3′	
*PKB (AKT1)*	Forward	5′‐TAGGCATCCCTTCCTTACAG‐3′	NM033230
	Reverse	5′‐GCCCGAAGTCCGTTATCT‐3′	
*ICAM‐1*	Forward	5′‐AGCGACATTGGGGAAGACAG‐3′	NM_012967.1
	Reverse	5′‐GGGAAGTACCCTGTGAGGTG‐3′	

### 2.7 Statistical Analysis

We utilized mean ± standard errors to represent quantitative variables. To examine whether the sample data followed normal distribution, we conducted the Kolmogorov‐Smirnov (K‐S) test. We performed a one‐way analysis of variance (ANOVA) to evaluate the differences in means among the studied groups. After identifying statistically significant results, a post hoc Bonferroni correction test was performed to identify differences between group means. All statistical analyses were conducted using SPSS version 20 (Chicago, IL, USA). We The threshold for statistical significance was set at *p* < 0.05.

## 3 Results

### 3.1 Effect of CAY10500 on the Expression of TNFR1

**Figure 1:**
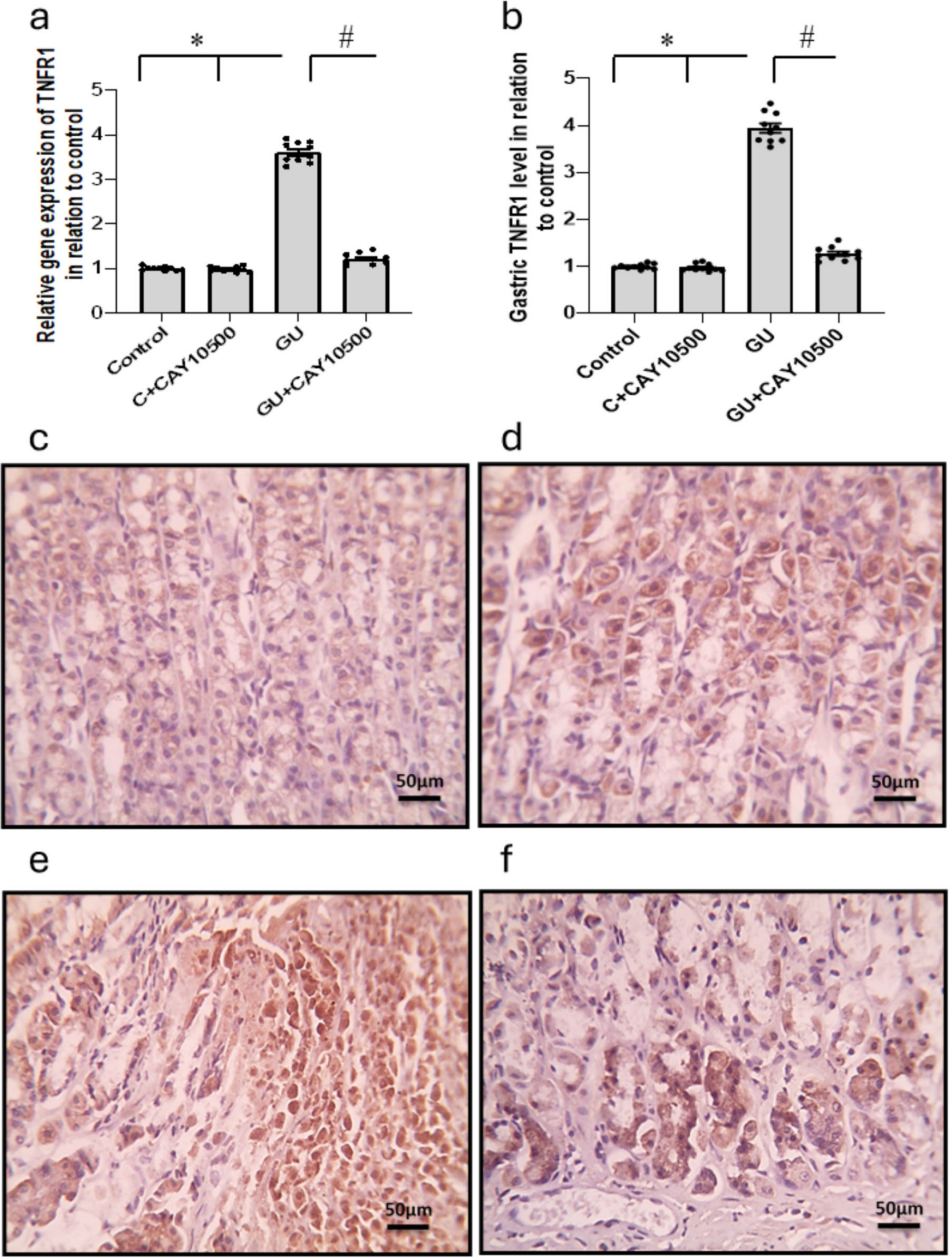
Effect of GU and 1 mg/kg CAY10500 on gene expression of TNFR1 (a) and its protein level in gastric tissues (b). Gastric sections stained with anti‐TNFR1 in the control group (c), control group treated with CAY10500 (d), GU group (e), and GU treated with CAY10500 (f). Scale bar 50 μm. The difference between groups was assessed by ANOVA, followed by the post hoc Bonferroni correction test upon establishing statistical significance. * Significant difference as compared with the control group at *p* < 0.05. ^#^ Significant difference as compared with GU group at *p* < 0.05. GU, gastric ulcer; TNFR1, tumor necrosis factor‐α type 1 receptor.

The examination of gastric tissues showed a considerable 3.61‐ and 3.95‐fold rise in TNFR1 gene expression and its gastric protein levels in the GU group compared to the control group. Treatment of GU with CAY10500 significantly reduced these effects to 1.22‐ and 1.27‐fold increases, respectively, as compared with the control group. Treatment with CAY10500 did not affect the control group. TNFR1 immunostaining supported these findings, demonstrating increased gastric TNFR1 expression in rats with GU. Additionally, the treatment reduced gastric TNFR1 immunostaining in the GU group without affecting the control group (see Figure 1).

### 3.2 Effect of CAY10500 on GU

**Figure 2:**
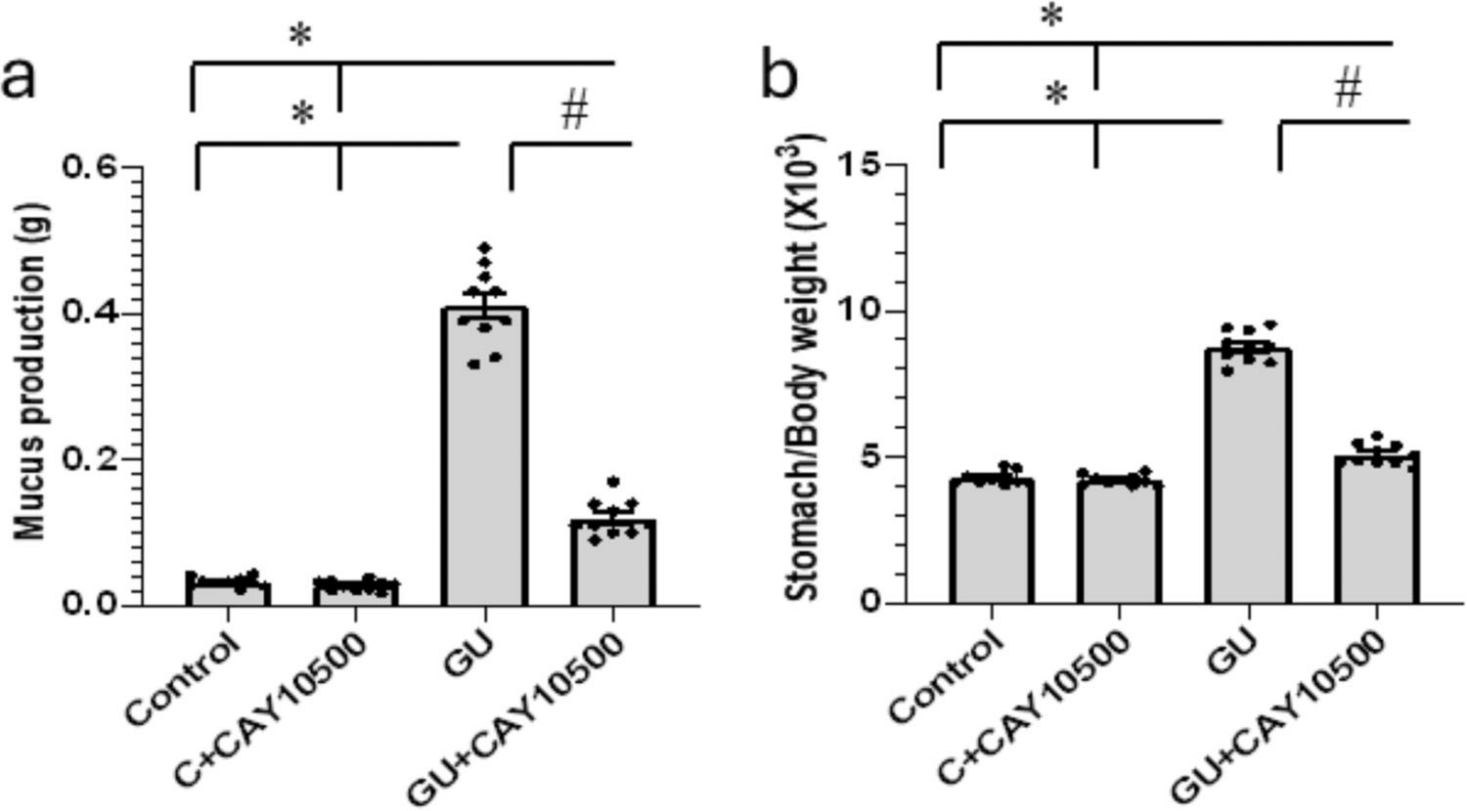
The effect of GU and 1 mg/kg CAY10500 on mucus production (a) and stomach/body weight ratio (b). The difference between groups was assessed by ANOVA, followed by the post hoc Bonferroni correction test upon establishing statistical significance. * Significant difference as compared with the control group at *p* < 0.05. ^#^ Significant difference as compared with GU group at *p* < 0.05. GU, gastric ulcer.

Indomethacin‐treated rats showed signs of developing GU, identified by a significant 13.67‐fold increase in mucus production (0.41 ± 0.17 g) and a twofold rise in the stomach/body weight ratio (8.76 ± 0.17) compared to the control group (0.032 ± 0.002 g and 4.28 ± 0.072, respectively). However, using CAY10500 led to a 71% decrease in mucus production (0.12 ± 0.008 g) and an improvement in the stomach/body weight ratio (5.08 ± 0.116) compared to the rats with GU (Figure 2).

### 3.3 Effect of CAY10500 on Gastric Tissue Structure

**Figure 3:**
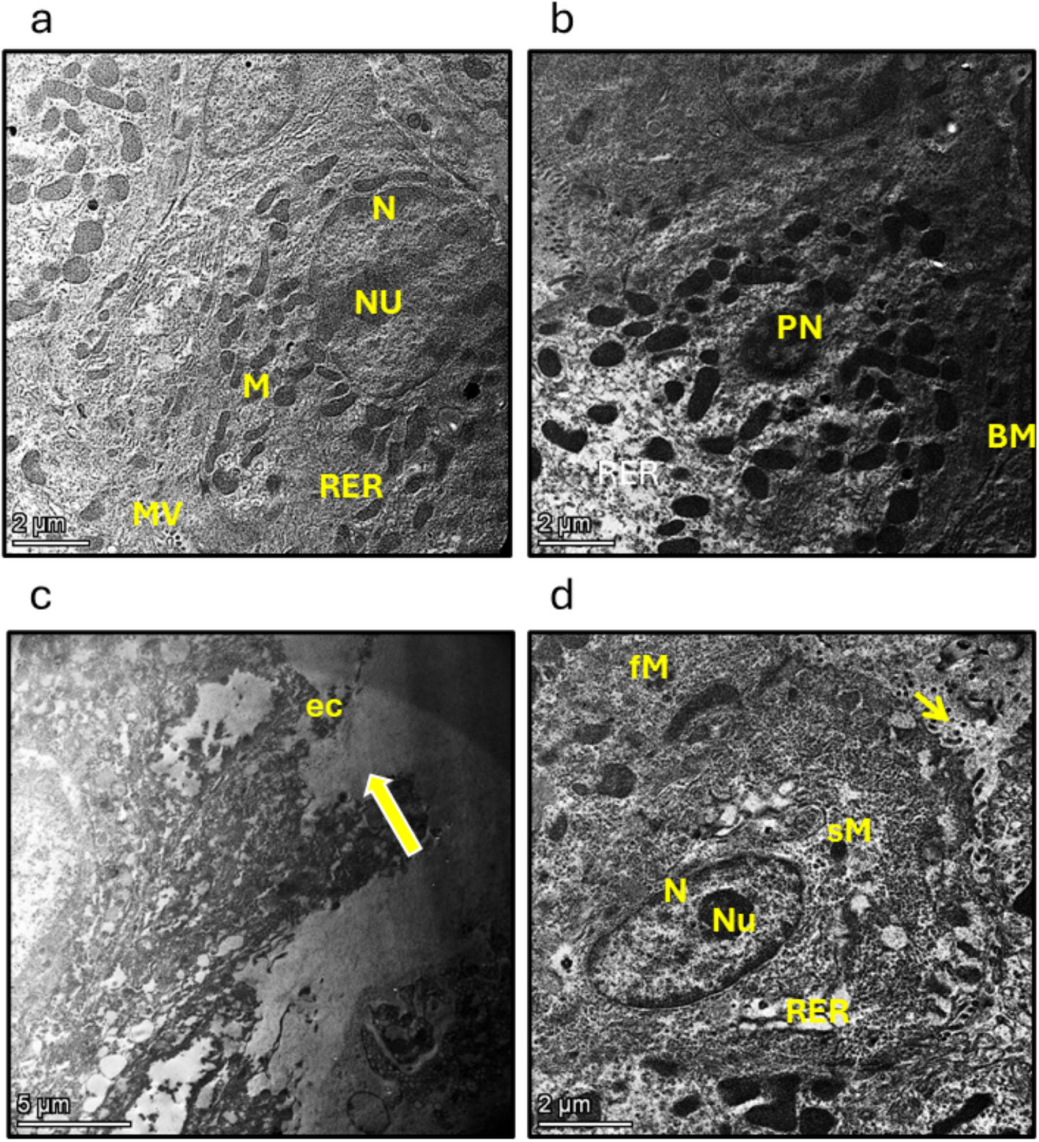
Representative transmission electron microscope micrograph of stomach from different treatment groups. (a) The control group showed normal ultrastructure of lining cuboidal cells with microvillous projection (mv), normal cytoplasmic mitochondria (m), RER and nucleus (n) with prominent nucleolus (nu). (b) The control group treated with CAY10500 showing nuclear pyknosis (PN), dilated RER (RER), partial widen in tight junction (thin arrow) between epithelial cells, intact basement membrane (BM). (c) The gastric ulcer group showing extensive necrosis with complete loss of normal ultrastructure of epithelial nucleus and cytoplasmic organelles, loss of tight junction, disruption of cell membranes (thick arrows), massive cell exfoliation (ec). (d) The gastric ulcer group treated with CAY10500 showing abundant dilated RER (RER), irregular shaped, shrunken (Sm) and fragmented (fm) with reduction in number of mitochondria, decrease in nuclear size (n) with prominent nucleolus (nu) and partial loss of epithelial microvilli (thin arrow). The scale bar equal 5 µm.

In the representative transmission electron microscope micrograph of the stomach from the control group, observations revealed the presence of normal ultrastructure of lining cuboidal cells with microvillous projections (mv), normal cytoplasmic mitochondria (m), RER, and a nucleus (n) with a prominent nucleolus (nu). In the micrographs from the control group treated with CAY10500, nuclear pyknosis (PN), dilated RER, partial widening in the tight junction (thin arrow) between epithelial cells, and intact basement membrane (BM). Conversely, the micrographs from the GU group showed extensive necrosis, complete loss of normal ultrastructure of the epithelial nucleus and cytoplasmic organelles, loss of tight junction, disruption of cell membranes (thick arrows), and massive cell exfoliation. Lastly, the micrographs from the GU group treated with CAY10500 exhibited abundant dilated RER, irregularly shaped, shrunken (Sm), and fragmented (fm) cytoplasmic mitochondria, a reduction in the number of mitochondria, a decrease in nuclear size (n) with a prominent nucleolus (nu), and partial loss of epithelial microvilli (thin arrow) (Figure 3).

**Figure 4:**
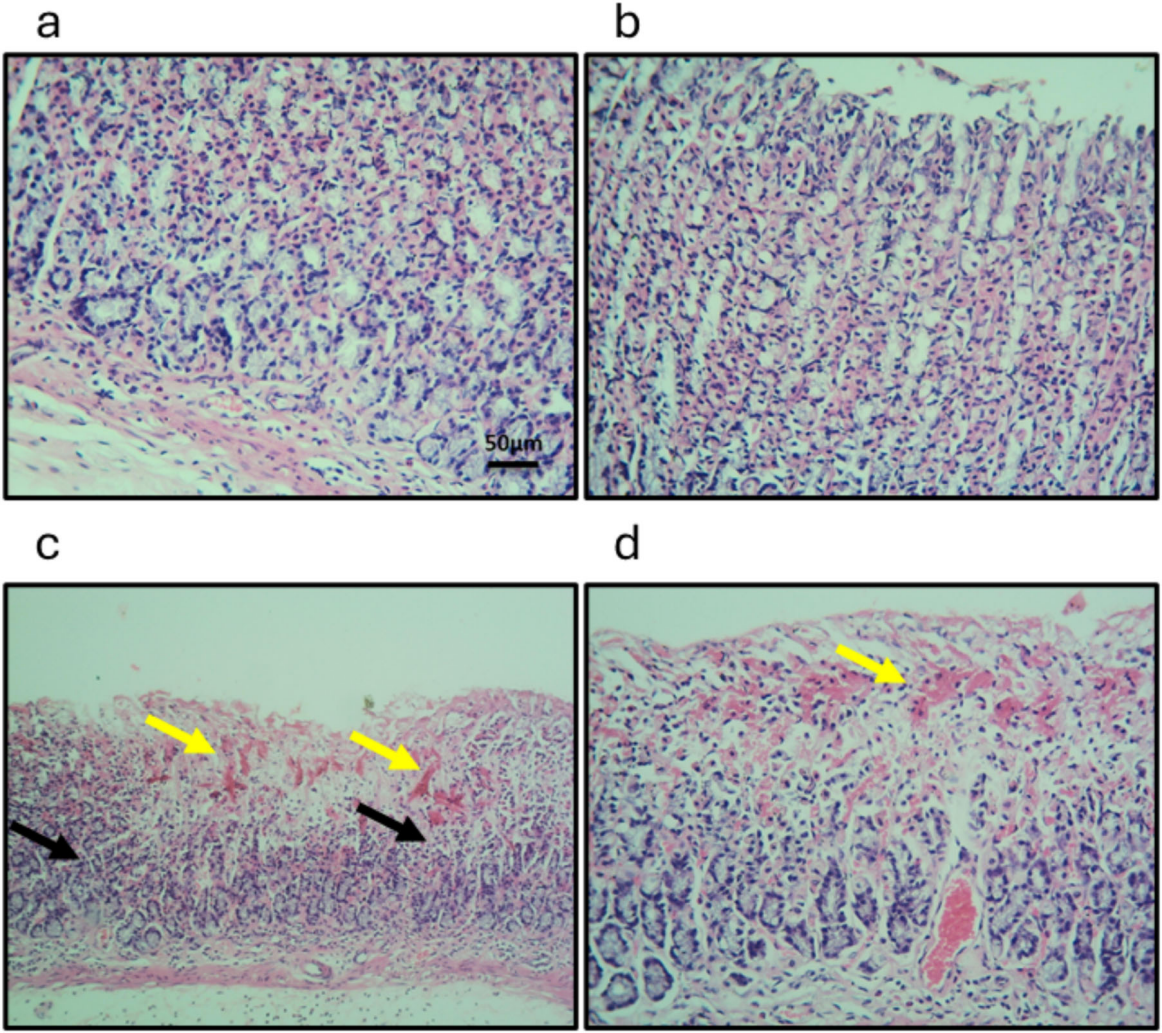
Gastric sections stained with hematoxylin/eosin control group (a), control group treated with CAY10500 (b), GU group (c) and GU treated with CAY10500 (d). Yellow arrows represented focal mucosal inflammation and black arrows represented extensive mucosal necrosis. Scale bar 50 μm. GU, gastric ulcer.

The microsections derived from the GU group, stained with hematoxylin and eosin, exhibited focal mucosal inflammation (highlighted by the yellow arrow) and extensive mucosal necrosis (indicated by the black arrows) compared to the control group. Furthermore, the administration of CAY10500 partially restored the mucosal layer structure in the subjects treated with indomethacin, suggesting its potential protective efficacy (Figure 4).

### 3.4 Effect of CAY10500 Treatment on GU‐Induced Alteration in VEGF Expression

**Figure 5:**
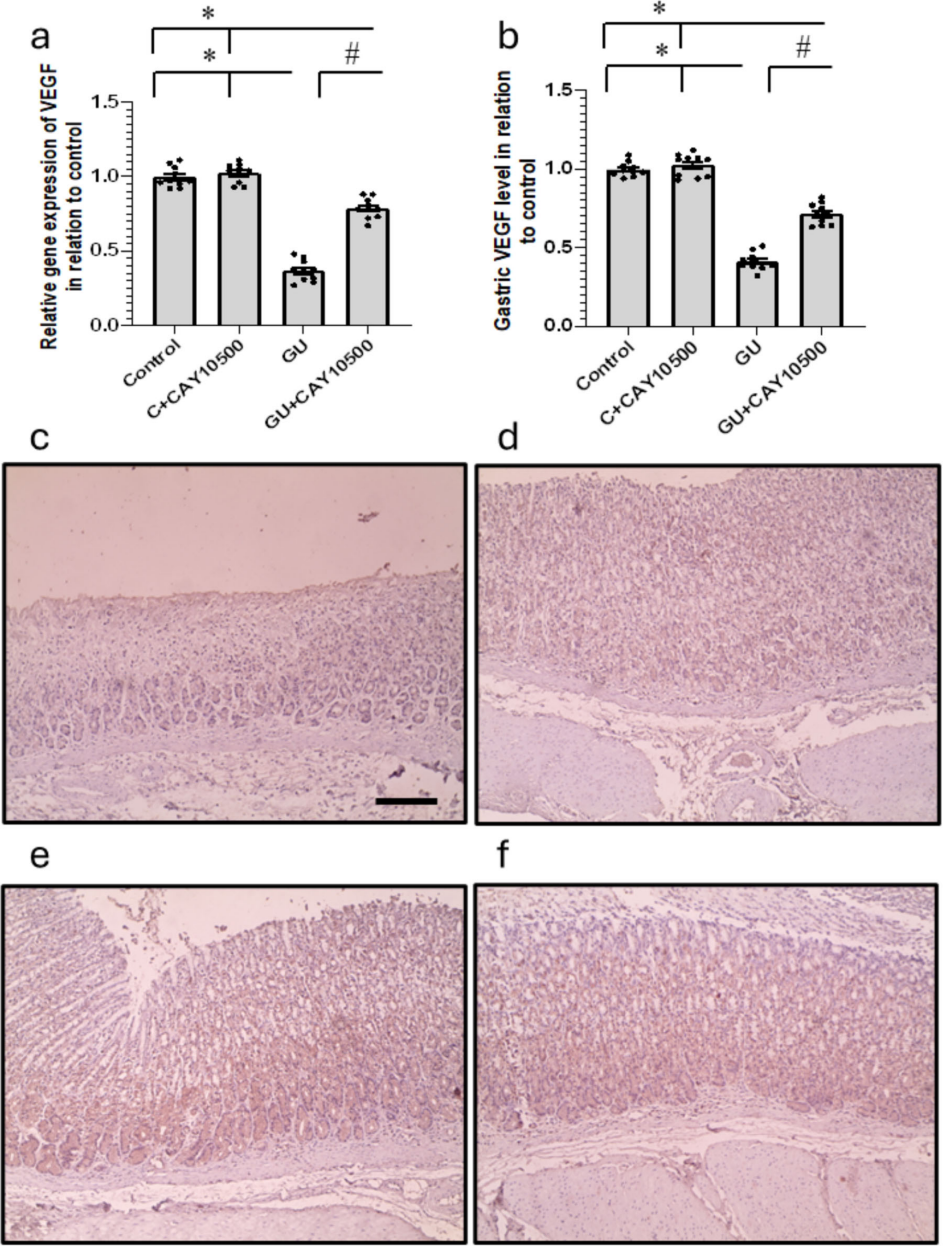
Effect of GU and 1 mg/kg CAY10500 on gene expression of VEGF (a) and its protein level in gastric tissues (b). Gastric sections stained with anti‐VEGF in the control group (c), control group treated with CAY10500 (d), GU group (e), and GU treated with CAY10500 (f). Scale bar 100 μm. The difference between groups was assessed by ANOVA, followed by the post hoc Bonferroni correction test upon establishing statistical significance. * Significant difference as compared with the control group at *p* < 0.05. ^#^ Significant difference as compared with GU group at *p* < 0.05. GU, gastric ulcer; VEGF, vascular endothelial growth factor.

Analysis of stomach tissue revealed a significant 63% and 59% reduction in VEGF gene expression and protein levels in the stomach of the GU group compared to the control group. These effects were significantly elevated following the administration of CAY10500. The findings were supported by VEGF immunostaining, which indicated a noticeable reduction in stomach VEGF expression in GU rats. Furthermore, treatment with CAY10500 increased stomach VEGF immunostaining in the GU group while not affecting the control group (Figure 5).

### 3.5 Effect of CAY10500 on GU‐Induced Downregulation of ERK

**Figure 6:**
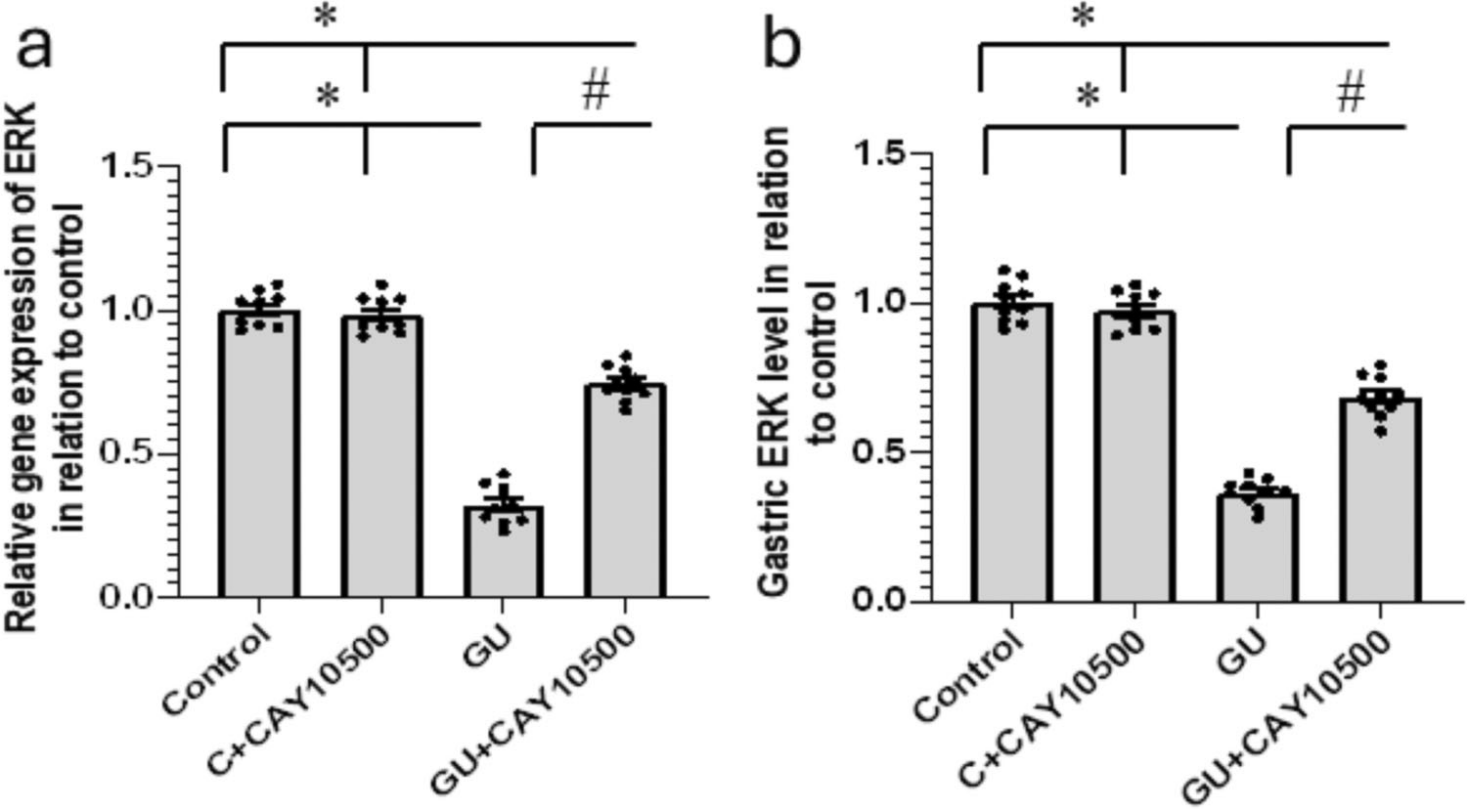
Effect of GU and 1 mg/kg CAY10500 on gene expression of ERK (a) and its gastric protein levels (b). The difference between groups was assessed by ANOVA, followed by the post hoc Bonferroni correction test upon establishing statistical significance. * Significant difference as compared with control group at *p* < 0.05. ^#^ Significant difference as compared with GU group at *p* < 0.05. ERK, extracellular signal‐regulated kinase; GU, gastric ulcer.

In the context of GU, the ERK gene was downregulated by a significant 68% and 63% reduction in gene expression and gastric protein levels compared to the control group. Notably, the administration of CAY10500 to GU rats reversed these effects in the GU group without affecting the control group (Figure 6).

### 3.6 Effect of CAY10500 on GU‐Induced Downregulation of PI3K and AKT

**Figure 7:**
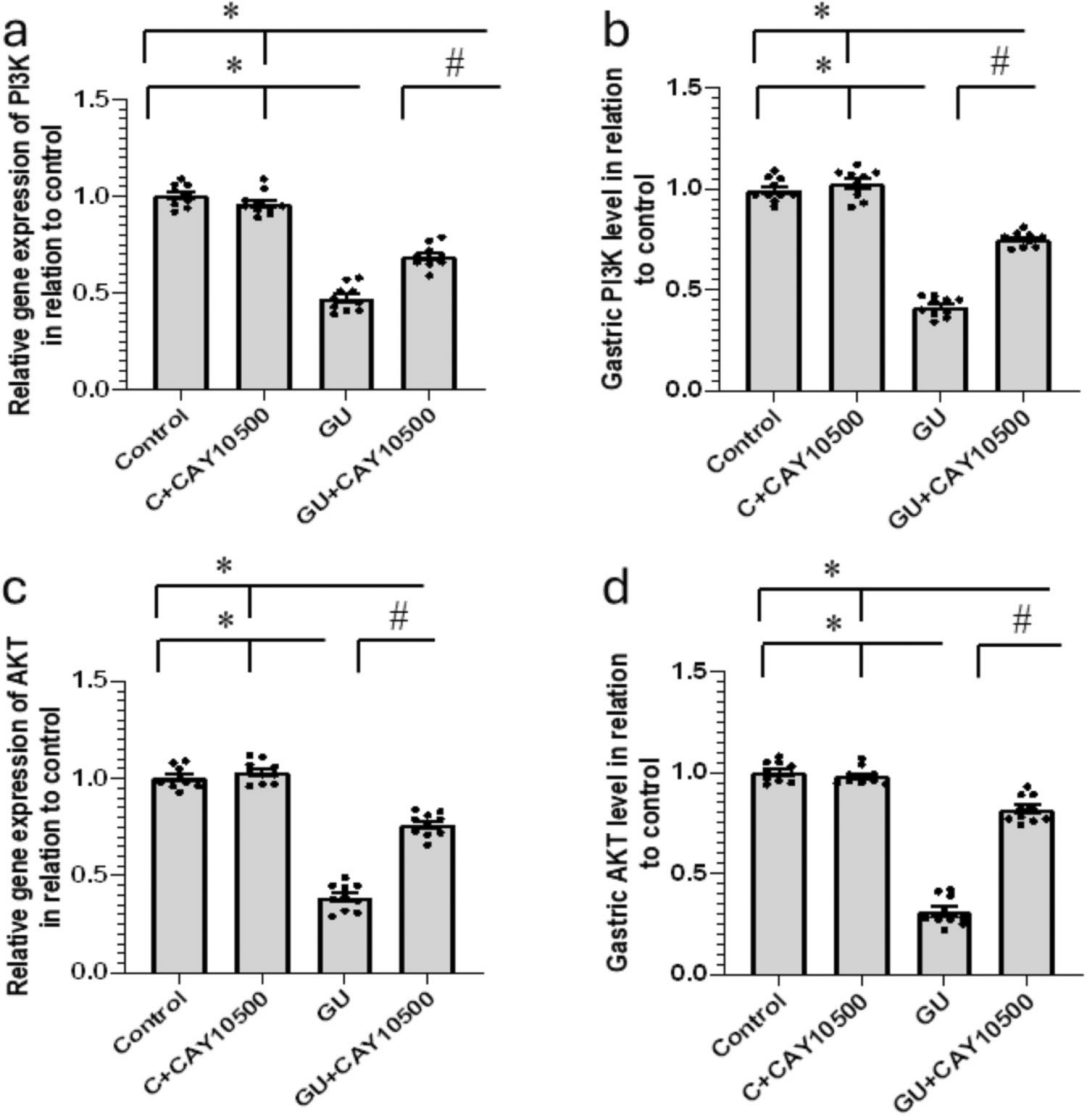
Effect of GU and 1 mg/kg CAY10500 on gene expression of PI3K (a) and AKT (c) and the gastric protein levels of PI3K (b) and AKT (d). The difference between groups was assessed by ANOVA, followed by the post hoc Bonferroni correction test upon establishing statistical significance. * Significant difference as compared with control group at *p* < 0.05. ^#^ Significant difference as compared with GU group at *p* < 0.05. GU, gastric ulcer; PI3K, phosphatidylinositol‐3 kinase; AKT, protein kinase B.

In the GU group, the gene expression of PI3K and AKT was significantly reduced by 53% and 61%, respectively. Furthermore, the levels of PI3K and AKT proteins in the gastric tissues exhibited a 58% and 69% decrease compared to the control group. Notably, the administration of CAY10500 to GU rats reversed these effects, as illustrated in Figure 7, with no discernible impact on the control group (Figure 7).

### 3.7 Effect of CAY10500 Treatment on GU‐Induced Alteration in ICAM‐1 Expression

**Figure 8:**
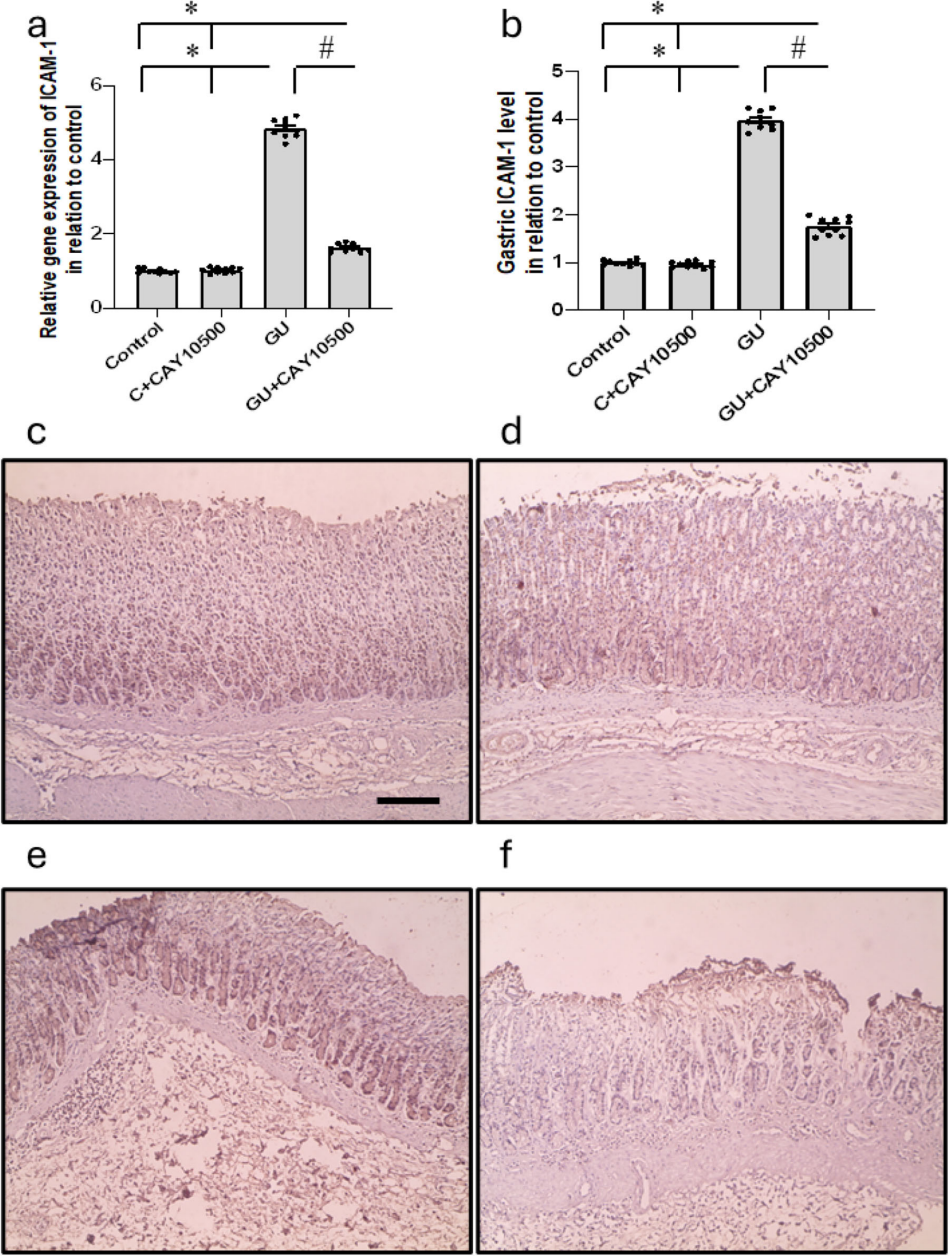
Effect of GU and 1 mg/kg CAY10500 on gene expression of ICAM‐1 (a) and its protein level in gastric tissues (b). Gastric sections stained with anti‐ICAM‐1 in the control group (c), control group treated with CAY10500 (d), GU group (e), and GU treated with CAY10500 (f). Scale bar 100 μm. The difference between groups was assessed by ANOVA, followed by the post hoc Bonferroni correction test upon establishing statistical significance. * Significant difference as compared with the control group at *p* < 0.05. ^#^ Significant difference as compared with GU group at *p* < 0.05. GU, gastric ulcer; ICAM‐1, intercellular adhesion molecule‐1.

Examination of stomach tissue revealed a significant 3.84‐ and 3.97‐fold increase in ICAM‐1 gene activity and protein levels in the GU group compared to the control group. These effects were notably reduced following the administration of CAY10500. The findings were corroborated by ICAM‐1 immunostaining, indicating a substantial elevation in stomach ICAM‐1 expression in GU rats. Moreover, the administration of CAY10500 resulted in a reduction of stomach ICAM‐1 immunostaining in the GU group to the level of 1.62‐ and 1.76‐fold, respectively, while having no impact on the control group (Figure 8).

## 4 Discussion

GU is a well‐established condition associated with various complications, including bleeding, perforation, and obstruction. The global frequency of GU has been increasing, primarily due to the widespread utilization of NSAIDs and alcohol [23]. Multiple animal models are employed to induce GU in rats. In our study, we opted to administer a single oral dose of indomethacin to fasting rats, a method previously shown to cause inflammation, ulceration, and bleeding in the stomach [24]. Our findings indicated significant changes in the shape of the stomach after administering indomethacin. These changes included bleeding and widespread ulcerated areas. Additionally, there was a substantial increase in mucus production and the ratio of stomach weight to body weight. The higher ratio of stomach weight to body weight suggested that the stomach was swollen due to inflammation and bleeding, and the body weight was reduced due to decreased food consumption and wasting caused by pain and discomfort. Moreover, when stomach sections from GU rats were examined under a microscope and stained with H/E, a noticeable localized inflammation of the stomach lining and extensive tissue death were observed. Furthermore, when microsections from the GU group were analyzed using an electron microscope, extensive tissue death, complete loss of the normal structure of the cell nucleus and organelles, disruption of tight junctions, cell membrane damage, and significant cell shedding were observed. This comprehensive examination provided valuable insights into the impact of indomethacin on the health and function of the stomach.

Pharmaceutical treatments for GU disorders can result in significant expenses and notable adverse effects. Our primary aim was to thoroughly investigate the potential therapeutic advantages of inhibiting TNFR1 for GU conditions. We specifically focused on assessing the effectiveness of CAY10500 as a targeted and competitive TNFR1 inhibitor for GU disorders. Following the administration of CAY10500, we observed a remarkable improvement in stomach structure, reduced mucus production, and decreased stomach‐to‐body ratio. Further micro‐section analysis using H/E staining revealed a substantial enhancement in gastric tissue structure. Finally, after examination under an electron microscope, the micrographs from the GU group treated with CAY10500 exhibited a reduction in the number of mitochondria, a decrease in nuclear size with a prominent nucleolus, and partial loss of epithelial microvilli. This encouraged us to progress to the next phase, where we aimed to uncover the mechanism of action of CAY10500 in treating GU disorders.

VEGF was initially identified as a vascular permeability factor due to its capacity to enhance blood vessel permeability. It plays a pivotal role in regulating angiogenesis, forming new blood vessels from existing ones. VEGF primarily functions as a mitogen, stimulating the growth and division of endothelial cells, as its receptors are predominantly present in these cells [25]. VEGF attaches to distinct receptors, specifically VEGF‐R1 and VEGF‐R2, predominantly found in endothelial cells. When VEGF interacts with these receptors, it triggers the phosphorylation of different intracellular proteins. This phosphorylation process sets off signal transduction pathways that lead to the proliferation, movement, and development of new microvascular tubes in endothelial cells, ultimately promoting angiogenesis [26]. Research demonstrating that accelerating angiogenesis in granulation tissue by administering exogenous VEGF significantly hastens the healing of gastric and duodenal ulcers in experimental rat models highlights the importance of angiogenesis in ulcer healing [27]. Furthermore, applying VEGF locally along with angiopoietin‐1 cDNAs or serum response factor (SRF) plasmids has significantly sped up the healing of GU and enhanced the quality of mucosal regeneration in ulcer scars. On the other hand, administering a neutralizing anti‐VEGF antibody has been observed to reduce the accelerated healing of ulcers resulting from these treatments [28]. However, we found a significant reduction in the expression of VEGF in the GU group, which was increased by treatment with CAY10500.

The ERK signaling pathway has been previously confirmed to play a crucial role in the recovery of GU. Notably, the activity of Erk1/2 was significantly elevated in epithelial cells located at the edge of the ulcer. Several investigations have shown that the increased activity of ERK is strongly associated with a significant rise in cell proliferation [29, 30]. The increased expression of ERK indicates a more significant growth of gastric mucosal epithelial cells, indicating that the growth of these cells might assist in replacing the damaged mucosal tissue and help restore the injured mucosa [30]. Furthermore, it has been observed that ERK is implicated in EGF‐induced cell proliferation and VEGF‐induced angiogenesis. Moreover, the inhibition of EGF‐R kinase resulted in a notable delay in healing GU through the substantial blockade of ERK [31]. However, the expression of ERK was considerably diminished in the group afflicted with GU, and this reduction was reversed following treatment with CAY10500.

The targets of treatments aimed at GU are distributed across various signaling pathways, primarily associated with inflammation. Among these pathways, the PI3K‐Akt signaling pathway holds particular significance. Activation of the PI3K‐Akt pathway plays a crucial role in cell proliferation and movement, significantly influencing ulcer healing and re‐epithelialization. This pathway carries substantial implications for the processes involved in resolving GU [32]. The AKT/PKB pathway is essential for controlling GU function. It consists of three forms: PKBα (AKT1), PKBβ (AKT2), and PKBγ (AKT3). This pathway is vital in regulating different cellular reactions, such as the cell cycle, cell growth, autophagy, and apoptosis in the GU system [33]. The AKT/PKB pathway regulates the interplay between autophagy and apoptosis in the GU. Its activation primarily governs the PI3K‐Akt signaling pathway, pivotal for cellular proliferation and motility within the GU. Prior research emphasized that dysregulation of the AKT/PKB pathway can disrupt autophagic flux and apoptosis, highlighting its significance in preserving proper cellular function within the GU context [34]. We found reduced expression of PI3K/PKB in GU, which was ameliorated by treatment with CAY10500. However, no previous study illustrated the ability of CAY10500 to regulate ERK/PKB in GU.

ICAM‐1 belongs to the immunoglobulin superfamily and is an essential cell adhesion molecule in different cell types, such as leukocytes and endothelial cells. Its primary role is to facilitate the movement of leukocytes through the endothelium, particularly during inflammation [35]. In the presence of inflammatory cues, the vascular endothelium increases the expression of ICAM‐1, which aids in the attraction and attachment of leukocytes to the endothelium during vascular inflammation. Irregular expression of ICAM‐1 has been linked to the onset of various vascular disorders [36]. ICAM‐1 is crucial in regulating cell‐cell interactions and modulating inflammatory responses. In the context of GU, heightened levels of ICAM‐1 in mucosal tissues, in conjunction with gastric acid, have been identified as primary contributors to ulcer recurrence. These elevated levels also promote the infiltration of neutrophils into injured gastric tissue, exacerbating the inflammatory response [37]. The expression of ICAM‐1 in the pathogenesis of GU has been shown to decrease with therapeutic agents targeting GU, indicating the critical role of ICAM‐1. This demonstrates the potential of ICAM‐1 as a therapeutic target for treating gastrointestinal disorders [38]. However, in the current study, we discovered a significant increase in the expression of ICAM‐1 in GU rats that was reduced by treatment with CAY10500.

## 5 Conclusion

Our investigation of CAY10500 demonstrates the efficacy of the specific TNFR1 inhibitor in mitigating experimentally induced GU in rats. Its effectiveness stems from its mechanism of action, which centers on the inhibition of TNFR1. Additionally, CAY10500 has exhibited the capacity to promote neovascularization by upregulating the expression of VEGF. It has also demonstrated proficiency in inhibiting cell adhesion by reducing the expression of ICAM‐1. Furthermore, it activates the cell proliferation pathway by upregulating the expression of PI3K/AKT and ERK, leading to the regeneration of mucosal cells.

## Author Contributions

H.M.H., F.E.A., O.B., and M.M.H.A. performed the biochemical analysis. A.A. and R.A. performed the animal experiments. O.B. and M.M.H.A. performed the pathological and immunohistochemistry analysis, and H.M.H., F.E.A., and O.B. performed the statistical analysis. M.M.H.A. came up with the study's concept and supervised the work. H.M.H., F.E.A., A.A., and R.A. helped develop and design the present study. All authors contributed to the writing of the manuscript and approved the final version.

## Ethics Statement

The research protocol was approved by the Research Ethics Committee of the Faculty of Pharmacy, Delta University for Science and Technology (FPDU‐REC), and approval No. (FPDU19/2022) was granted.

## Consent

The authors have nothing to report.

## Conflicts of Interest

The authors declare no conflicts of interest.

## Data Availability

The data that supports the findings of this study are available from the corresponding author upon reasonable request.
